# Supplementary material for: Impact of Baking Powder and Leavening Acids on Batter and Pound Cake Properties
Source: Foods. 2023 Feb 23;12(5):946. doi: 10.3390/foods12050946 (PMC10001083; doi:10.3390/foods12050946)
Supplement: Supplementary file 1 [file foods-12-00946-s001.zip › foods-2207135-supplementary.pdf]

Experimental Design Process

Using Statgraphics Centurion 19 software (Statgraphics Technologies, Inc, The Plains, VA, USA):

Response Surface Design Attributes

Design class: Response Surface
Design name: Central composite design: 2^2 + star
Design characteristic: Rotatable

Base Design
Number of experimental factors: 2
Number of blocks: 1
Number of responses: 8
Number of runs: 10, including 2 centerpoints per block
Error degrees of freedom: 4
Randomized: Yes

Table S1: Experimental factors established

Factors	*Low*	*High*	*Units*	*Continuous*
**Baking Powder**	1.5	4	%	Yes
**SAPP 10**	0.1465	0.8535	%	Yes

Table S2: Parameters selected as responses

Responses	*Units*
**Batter Specific Volume**	cm^3/g
**Batter Porosity**	%
**Batter pH**	
**Cake Specific Volume**	cm^3/g
**Cake Porosity**	%
**Cake Total Height**	cm
**Cake Base Height**	cm
**CO_2 Liberated**	g

Regression equations of responses

The regression equations of the fitted model for of each parameters. are as follows

Equation S1:

Batter Specific Volume = 1.09996 - 0.0149914*Baking Powder - 0.0532656*SAPP 10 + 0.000651894*Baking Powder^2 + 0.012*Baking Powder*SAPP 10 + 0.0267134*SAPP 10^2

Equation S2:

Batter Porosity = 26.914 - 1.05446*Baking Powder - 3.71273*SAPP 10 + 0.043577*Baking Powder^2 + 0.853905*Baking Powder*SAPP 10 + 1.83131*SAPP 10^2

Equation S3:

Batter pH = 6.62981 + 0.0251323*Baking Powder + 0.267195*SAPP 10 - 0.00679585*Baking Powder^2 + 0.0293333*Baking Powder*SAPP 10 + 0.311869*SAPP 10^2

Equation S4:

Cake Specific Volume = 2.11108 + 0.347783*Baking Powder + 0.170823*SAPP 10 - 0.0559861*Baking Powder^2 - 0.0333333*Baking Powder*SAPP 10 + 0.00793157*SAPP 10^2

Equation S5:

Cake Porosity = 64.639 + 3.95326*Baking Powder + 0.311227*SAPP 10 - 0.591965*Baking Powder^2 - 0.338095*Baking Powder*SAPP 10 + 1.27168*SAPP 10^2

Equation S6:

Cake Total Height = 8.45468 + 1.55306*Baking Powder + 1.28345*SAPP 10 - 0.272205*Baking Powder^2 - 0.277905*Baking Powder*SAPP 10 - 0.459865*SAPP 10^2

Equation S7:

Cake Base Height = 5.37932 + 0.513094*Baking Powder + 0.102364*SAPP 10 - 0.132595*Baking Powder^2 + 0.380952*Baking Powder*SAPP 10 - 0.611027*SAPP 10^2

Equation S8:

CO_2 Liberated = 0.115448 + 0.0420097*Baking Powder + 0.00262938*SAPP 10 + 0.00164966*Baking Powder^2 + 0.000380952*Baking Powder*SAPP 10 + 0.0131244*SAPP 10^2

Table S3: Classification of significance levels in **Table 3** with respect to p-values with 95% confidence interval

**Significance Level**	**Range**
Not significant (NS)	> 0.05
+ (significant)	0.04 – 0.05
++ (very significant)	0.01 – 0.039
+++ (extremely significant)	0.000 – 0.009
